# Hemodynamic Changes following Aortic Valve Bypass: A Mathematical Approach

**DOI:** 10.1371/journal.pone.0123000

**Published:** 2015-04-16

**Authors:** Emilia Benevento, Abdelghani Djebbari, Zahra Keshavarz-Motamed, Renzo Cecere, Lyes Kadem

**Affiliations:** 1 Mechanical and Industrial Engineering Department, Concordia University, Montreal, Québec, Canada; 2 University Abou Bekr Belkaid, Tlemcen, Algeria; 3 Institute for Medical Engineering and Science, Massachusetts Institute of Technology, Cambridge, Massachusetts, United States of America; 4 Harvard-MIT Division of Health Sciences and Technology, Massachusetts Institute of Technology, Cambridge, Massachusetts, United States of America; 5 Department of Medicine, Laval University, Québec, Québec, Canada; 6 Department of Surgery, Division of Cardiac Surgery, McGill University, Montreal, Quebec, Canada; University of Washington, UNITED STATES

## Abstract

Aortic valve bypass (AVB) has been shown to be a viable solution for patients with severe aortic stenosis (AS). Under this circumstance, the left ventricle (LV) has a double outlet. The objective was to develop a mathematical model capable of evaluating the hemodynamic performance following the AVB surgery. A mathematical model that captures the interaction between LV, AS, arterial system, and AVB was developed. This model uses a limited number of parameters that all can be non-invasively measured using patient data. The model was validated using *in vivo* data from the literature. The model was used to determine the effect of different AVB and AS configurations on flow proportion and pressure of the aortic valve and the AVB. Results showed that the AVB leads to a significant reduction in transvalvular pressure gradient. The percentage of flow through the AVB can range from 55.47% to 69.43% following AVB with a severe AS. LV stroke work was also significantly reduced following the AVB surgery and reached a value of around 1.2 J for several AS severities. Findings of this study suggest: 1) the AVB leads to a significant reduction in transvalvular pressure gradients; 2) flow distribution between the AS and the AVB is significantly affected by the conduit valve size; 3) the AVB leads to a significant reduction in LV stroke work; and 4) hemodynamic performance variations can be estimated using the model.

## Introduction

Aortic stenosis (AS) is the most common valvular disease in the elderly population. Untreated symptomatic AS is associated with a poor prognosis and significant morbidity. Aortic valve replacement (AVR) is currently the standard of care for reducing the left ventricular overload and improving the quality of life of patients [1]. However, a significant proportion of patients (around 30% to 60%) are not referred to AVR because they fall within the category of high-risk patients [2,3,4]. This is typically because of comorbidities, severely calcified aorta (porcelain aorta) or narrow LVOT (<18 mm) [5]. Transcatheter aortic valve replacement (TAVI) has recently emerged as a good option for such inoperable patients. However, TAVI might also be not feasible in all inoperable patients, such as those with porcelain aorta, bicuspid aortic valve, ostial encroachment or small LVOT, leaving a sub-population of ‘no-option’ patients [6,7]. Furthermore, TAVI and AVR appear to have similar major stroke rates at 30 days and 1 year (p = 0.20 and p = 0.07, respectively). However, when strokes and transient ischemic events are considered, AVR showed significantly better results compared to TAVI both at 30 days and 1 year (p = 0.04) [8]. As a consequence, some investigators suggested the use of aortic valve bypass (AVB) as an alternative to TAVI or for patients with contraindications to both AVR and TAVI [9,10,11].

In AVB, also called apico-aortic bypass, the AS and the aorta are never manipulated and a second outflow tract for blood flow during systolic phase is created by inserting a valved conduit at the level of the left ventricle apex. This leads to a double outlet ventricle [12]. The outflow conduit is connected to the descending thoracic aorta. The advantages of this approach are: 1) the calcified aortic valve and the aorta are never manipulated; 2) it is less subjected to patient-prosthesis mismatch; 3) there is no risk of damage to the ascending aorta or obstruction of the coronary arteries; 4) it potentially leads to less risks of stroke compared to the AVR.

In AVB, several combinations of conduit size and conduit valve size can be used. An important clinical parameter to predict is the proportion of blood flow crossing the native stenotic valve *vs*. the bypass conduit. In addition, previous studies [13,14,15] showed that this flow distribution can be clinically determined using magnetic resonance imaging. Another important parameter to evaluate is the left ventricle stroke work, representing the work of the left ventricle during each heart beat, before and following the AVB surgery.

The objective of this study was to develop a mathematical model that can predict the variation in hemodynamic parameters (mainly transvalvular pressure gradients; flow distribution between the native aortic valve and the conduit) and left ventricle load following the aortic valve bypass surgery. The challenge was to develop a model solely based on non-invasive patient data, characteristics of the conduit (its diameter) and the hemodynamic performance of the conduit valve (its effective orifice area). We validated the model using existing clinical data and we provided results allowing a better understanding of the expected hemodynamic outcomes following the AVB surgery.

## Methods

**Fig 1:**
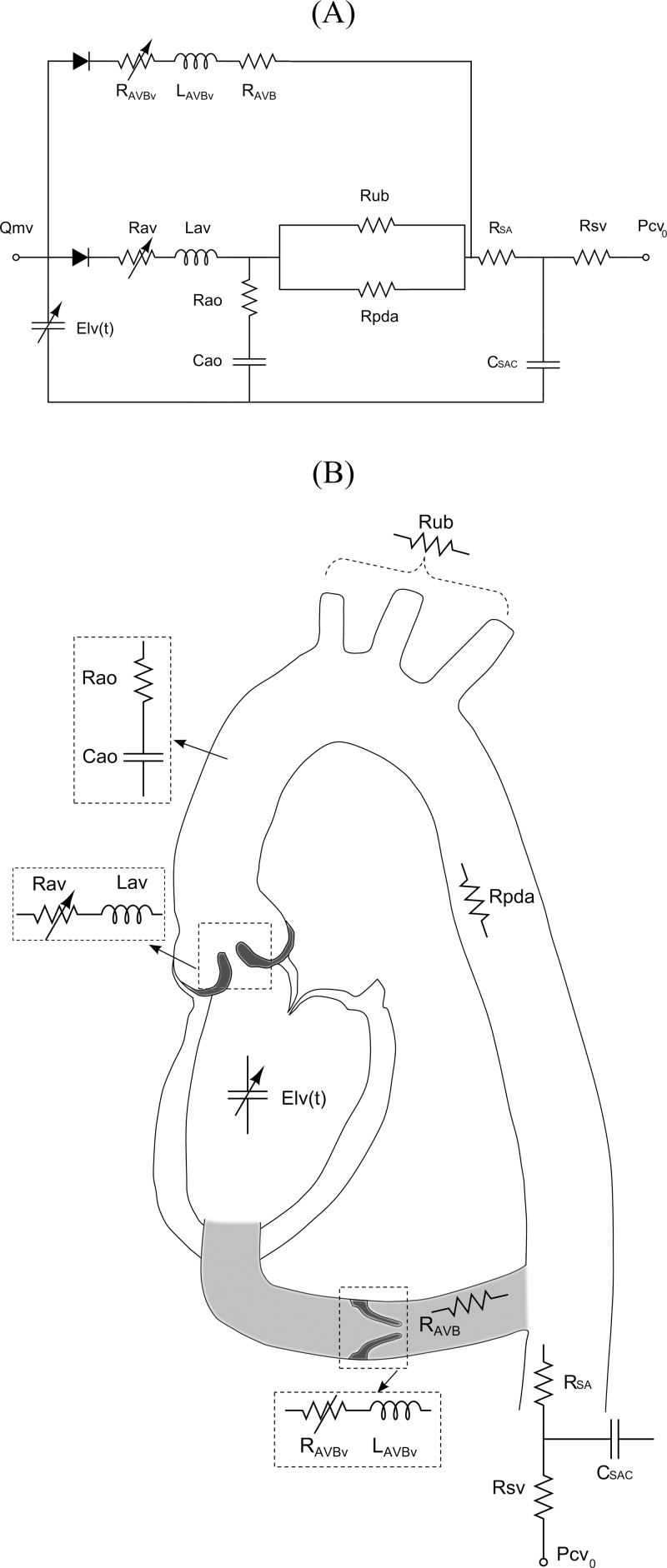
Schematic diagrams. (A) Electrical representation, (B) schematic representation of the lumped parameter model used to simulate left-sided heart in presence of aortic stenosis and/or apico-aortic conduit (please see Table 1 for all other parameters used in the lumped parameter model).

A schematic diagram of the lumped parameter model is presented in Fig 1. The code developed by Keshavarz-Motamed et al. [16,17] was modified to simulate the AVB. This model includes four different sub-models: 1) left ventricle (LV) model; 2) AS model; 3) systemic circulation model; and 4) AVB model. All parameters used in the lumped parameter model are listed in Table 1.

**Table 1:** Summarized cardiovascular parameters used to simulate all cases.

Description	Abbreviation	Value	Maximum error (%)*
**Ventricular parameters**			
Left-ventricular end-diastolic volume	LVEDV	150 ml	
Unstressed volume	V_0_	-15 ml	
Maximal elastance	E_max_	Adjusted for stroke volume 75 ml	
Time to maximal elastance	T_Emax_	0.24s	
**Valve parameters**			
Effective orifice area	EOA	From AS and AVB data	1.73
Energy loss coefficient	E_L_Co	From AS and AVB data	1.98
Variable resistance	R_av_	From AS and AVB data	2.16
Inductance	L_av_	From AS and AVB data	1.18
**Systematic circulation parameters**			
Aortic resistance	R_ao_	0.05 mmHg.s.ml^-1^	3.95
Aortic compliance	C_ao_	0.5 ml/mmHg	1.99
Systemic vein resistance	R_SV_	0.05 mmHg.s.ml^-1^	2.03
Systemic arteries and veins compliance	C_SAC_	2 ml/mmHg	2.03
systemic arteries resistance (including arteries, arterioles and capillaries)	R_SA_	0.8 mmHg.s.ml^-1^	1.97
Upper body resistance	R_ub_	Adjusted to have 15% of total flow rate in healthy case (Mcdonald, 1974)	1.98
Proximal descending aorta resistance	R_pda_	0.05 mmHg·s·ml^-1^	1.98
**Apico aortic conduit**			
AVB tube resistance	R_AVB_	0.05 mmHg·s·ml^-1^	1.99
**Output condition**			
Central venous pressure	P_CV0_	4 mmHg	
**Input condition**			
Mitral valve mean flow rate	Q_mv_	75 ml	
**Other**			
Constant blood density		1050 kg/m^3^	
Cardiac output	CO	5.2 l/min	
Heart rate	HR	70 beats/min	
Duration of cardiac cycle	T	0.857 s	

*Maximum error in computed ratio between AS and AVB flow rates from sensitivity analysis in response to independent variation (±30%) in each parameter

### Heart-Arterial Model

Heart function was described by time varying elastance as the following:

$$E(t)=\frac{P_{LV}(t)}{V(t)-V_{0}}$$ (1)

where P_LV_(t), V(t) and V_0_ are the LV pressure, the LV volume and the unloaded volume [18], respectively. The amplitude of E(t) was normalized with respect to maximal elastance E_max_, giving E_N_(t_N_) = E(t)/E_max_. Time was then normalized with respect to the time to attain peak elastance, T_Emax_ (t_N_ = t/T_Emax_). Note that normalized time-varying elastance curves E_N_(t_N_) have similar shapes in the normal human hearts with various inotropic situations or for diseased human hearts despite the existence of differences with regard to etiology of cardiovascular diseases [18,19].

$$E_{\max}E_{N}(t/T_{E\max})=\frac{P_{LV}(t)}{V(t)-V_{0}}$$ (2)

This normalized curve, represented as a Fourier series [19], can be described mathematically. Thus, when E_N_(t_N_) is given, the relation between *P*_*LV*_(*t*) and *V*(*t*) for any ventricle is determined. The ventricle is filled by a normalized physiological mitral flow waveform adjusted for the total required stroke volume, in this study 75 ml [16,17].

### Aortic Stenosis Model

The AS was modeled using the analytical formulation for the net pressure gradient (*TPG*_*net*_) across the stenotic valve during LV ejection introduced by Garcia et al. [20] This formulation expresses the instantaneous net pressure gradient across the AS as a function of the instantaneous flow rate and the energy loss coefficient.

$$TPG_{net}=\frac{2\pi \rho }{\sqrt{E_{L}Co}}\frac{\partial Q(t)}{\partial t}+\frac{\rho }{2E_{L}Co^{2}}Q^{2}(t)$$ (3)

and

$$E_{L}Co=\frac{(EOA)A}{A-EOA}$$ (4)

Where *E*_*L*_ *Co*, *EOA*, *A*, *ρ* and *Q* are the valvular energy loss coefficient, the effective orifice area, ascending aorta cross sectional area, the fluid density and the transvalvular flow rate, respectively. *E*_*L*_ *Co*, introduced by Garcia et al. [20], representing the ‘recovered *EOA*’, denotes valve effective orifice area adjusted for the area of the aorta at the level of sinotubular junction. Therefore, variable aortic valve resistance (*R*_*av*_) and constant aortic valve inductance (*L*_*av*_) (Fig 1) in the lumped parameter model are $\frac{\rho }{2E_{L}Co^{2}}Q$ and $\frac{\text{2}\pi \rho }{\sqrt{E_{L}Co}}$, respectively (Eq 3).

### Aortic Valve Bypass Model

In a normal heart, total blood flow ejected by the LV, after crossing the aortic valve, is redirected towards the upper-body. However, after the AVB surgery, a portion of the blood flow ejected by the LV goes towards the AS and the other portion towards the descending aorta through the bypass conduit. To take this into account in the model, a branch is placed in parallel to the AS (Fig 1). This branch simulates the flow bypassing the AS. Since, no formulation has been developed to express the net instantaneous pressure gradient through a conduit valve yet, we elected to use the same formulation as the one used for modeling the stenotic valve (Eq 3). Therefore, this branch includes a constant resistance for conduit tube (*R*_*AVB*_) (Table 1) plus a time-varying resistance ($R_{AVBv}=\frac{\rho }{2E_{L}Co^{2}}Q$), and an inductance ($L_{AVBv}=\frac{\text{2}\pi \rho }{\sqrt{E_{L}Co}}$) which together represent the net pressure gradient induced by the conduit valve (Eq 3). The EOA of the conduit valve was determined as follows: for a specific bioprosthetic valve size (between 19 and 29 mm), the reported EOAs of all the manufacturers listed in Pibarot et al. [21] were averaged. Yet a 17 mm valve can also be used for AVB surgery in adult patients. Therefore, the EOA for a conduit valve of 17 mm was extrapolated from the data reported in Pibarot et al [21].

### Computational Algorithm

Here, we provide a succinct description, see Keshavarz-Motamed et al. [16,17] for more details. The lumped model illustrated in Fig 1 was analyzed numerically by creating and solving a system of ordinary differential equations in Matlab Simscape (MathWorks, Inc). Fourier series representation of experimental normalized elastance curve for human adults introduced by Senzaki et al. [18] was used to generate a signal as an input into the main program. Simulation started at the onset of isovolumic contraction. The left ventricle volume *V*(*t*) was calculated using the left ventricle pressure *P*_*LV*_ and elastance values using Eq 1. The left ventricle is filled by a normalized physiological mitral flow waveform adjusted for the total required stroke volume. The left ventricle flow rate subsequently was calculated as the time derivative of the left ventricle volume. After few initial cycles, solution converged. Matlab’s “ode23t” trapezoidal rule variable-step solver was used to solve system of differential equations with the initial time step of 0.1 milliseconds. The convergence residual criterion was set to 10^–5^. All initial values including voltages and currents of capacitors and inductors were set to zero.

## Results

### Verification of the Lumped Parameter Model using *in vivo* Published Data

Verification of the model was done based on the study reported by Stauffer et al. [14] for different configurations of the AVB: 1) conduit size (diameter): 18 mm, conduit valve size (diameter): 19 mm; 2) conduit size: 20 mm, conduit valve size: 21 mm; and 3) conduit size: 22 mm, conduit valve size: 23 mm. Postoperative flow assessments using magnetic resonance imaging for these configurations show that 65% of the outflow is conducted from the LV apex to the conduit, while only 35% crosses the AS [14]. The blood flow proportion through a severe AS with two different severities (EOAs: 0.61 cm^2^ and 0.7 cm^2^) and different configurations of conduit were calculated using the lumped parameter model developed in this study and compared to the flow proportion from *in vivo* data. Data resulting from our mathematical simulations were consistent with Stauffer et al. findings (range of errors: 0.11% to 8.46%) (see Table 2) [14].

**Table 2:** Computed AS and AVB flow rate ratio in the presence of fixed severe AS (EOA = 0.7 cm2).

	AVB valve size (mm)						
AVB conduit size (mm)	17	19	21	23	25	27	29
18	AV: 40.25% AVB: 59.74%	AV: 36.57%* AVB: 63.43%	AV: 34.47% AVB: 65.53%	AV: 32.79% AVB: 67.21%	AV: 31.45% AVB: 68.56%	AV: 31% AVB: 69%	AV: 30.57% AVB: 69.43%
20	AV: 41.68% AVB: 58.32%	AV: 37.97% AVB: 62.03%	AV: 35.82%* AVB: 64.18%	AV: 33.99% AVB: 66.01%	AV: 32.43% AVB: 67.57%	AV: 31.86% AVB: 68.14%	AV: 31.32% AVB: 68.68%
22	AV: 42.70% AVB: 57.30%	AV: 39.02% AVB: 60.98%	AV: 36.84% AVB: 66.16%	AV: 34.96%* AVB: 65.04%	AV: 32.28% AVB: 66.72%	AV: 32.63% AVB: 67.36%	AV: 32% AVB: 68%
24	AV: 43.48% AVB: 56.52%	AV: 39.84% AVB: 60.16%	AV: 37.66% AVB: 62.33%	AV: 35.72% AVB: 64.28%	AV: 33.97% AVB: 66.03%	AV: 33.28% AVB: 66.72%	AV: 32.6% AVB: 67.4%
26	AV: 44.07% AVB: 55.93%	AV: 40.45% AVB: 59.55%	AV: 38.25% AVB: 61.73%	AV: 36.33% AVB: 63.67%	AV: 34.53% AVB: 65.47%	AV: 33.83% AVB: 66.17%	AV: 33.11% AVB: 66.9%
28	AV: 44.53% AVB: 55.47%	AV: 40.95% AVB: 59.05%	AV: 38.77% AVB: 61.23%	AV: 36.81% AVB: 63.19%	AV: 34.99% AVB: 65%	AV: 34.26% AVB: 65.74%	AV: 33.53% AVB: 66.47%

*Relative error in computed flow rates through native valve with EOA of 0.7 cm^2^ and AVB with combinations of 18–19, 20–21 and 22–23, compared to the results reported by Stauffer et al. (2011) are 4.49%, 2.34% and 0.11%, respectively. AV: aortic valve; AVB: aortic valve bypass

Stroke volume, heart rate and cardiac output are 75 ml, 70 beats/min and 5.2 l/min, respectively. AV: aortic valve, AVB: aortic valve bypass

### Pre-AVB Surgery: Simulation in the Presence of a Severe AS

**Fig 2:**
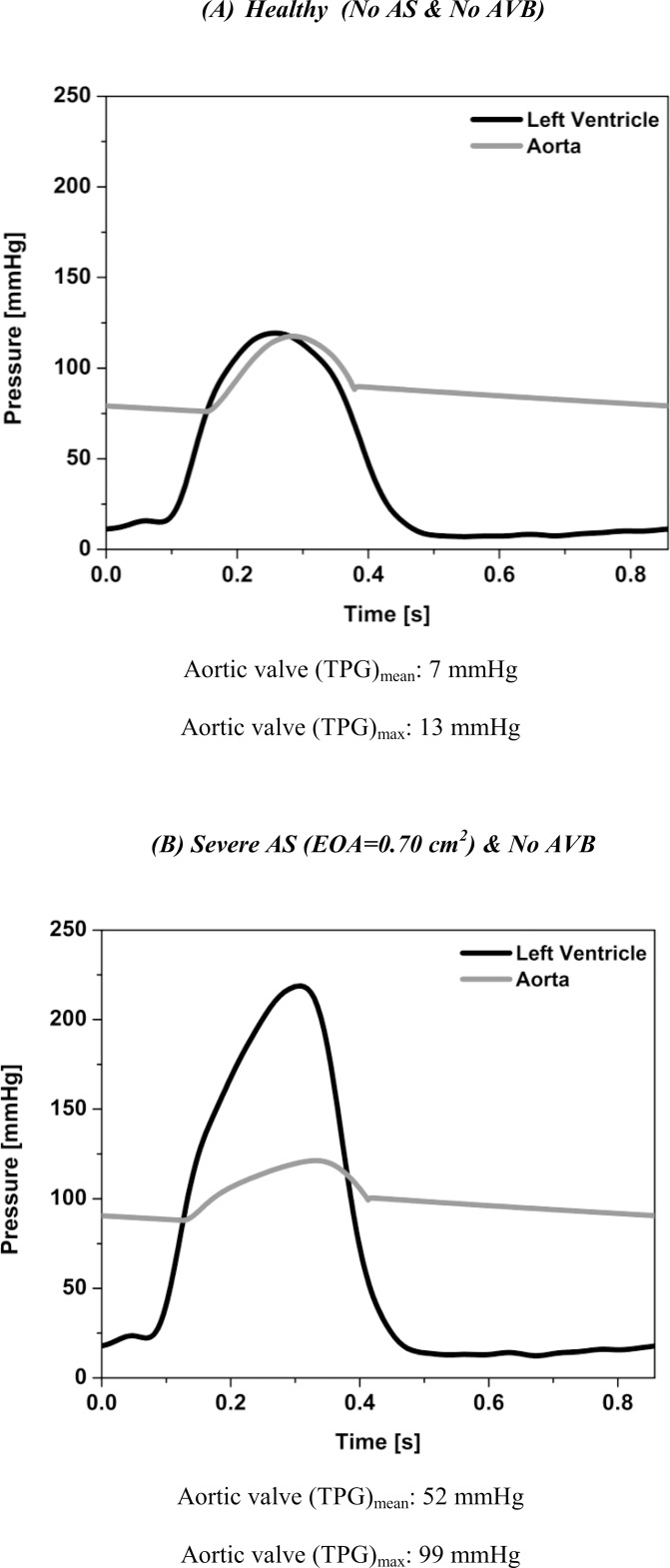
Simulated left ventricle and aorta pressures. (A) Healthy (No AS & No AVB), (B) severe AS (EOA = 0.7 cm^2^) & No AVB. Stroke volume, heart rate and cardiac output are 75 ml, 70 beats/min and 5.2 l/min, respectively.

Fig 2 shows the LV and aortic waveforms simulated using the current model for: 1) a healthy aortic valve (no AS: EOA = 4 cm^2^); 2) severe AS with EOA of 0.7 cm^2^. As expected, the presence of a severe AS induces very large transvalvular pressure gradients: TPG_max_ = 99 mmHg; TPG_mean_ = 52 mmHg. The results for an EOA of 0.7 cm^2^ are displayed because this EOA corresponds to the median value reported in the study of Lund et al. [5] for patients with AVB.

### Post- AVB Surgery: Simulation in the Presence of AS and AVB

**Fig 3:**
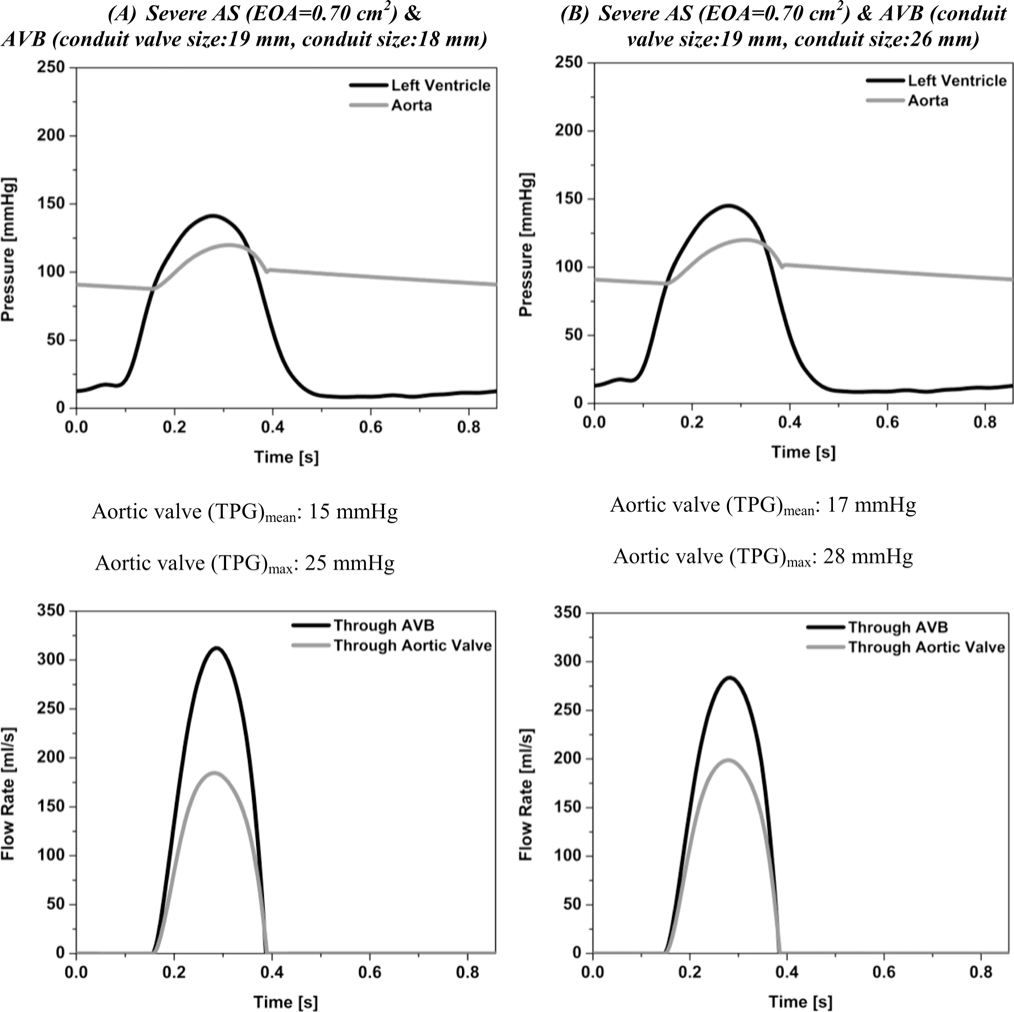
Simulated left ventricle and aorta pressures and flow distribution. (A) Severe AS (EOA = 0.7 cm^2^) & AVB (conduit valve size: 19 mm, conduit size: 18mm), (B) severe AS (EOA = 0.7 cm^2^) & AVB (conduit valve size: 19 mm, conduit size: 26mm). Stroke volume, heart rate and cardiac output are 75 ml, 70 beats/min and 5.2 l/min, respectively.

Fig 3a and 3b show the computed LV and aortic waveforms after the AVB surgery for the AS with an EOA of 0.7 cm^2^. Here the results for an AVB with a conduit valve size of 19 mm and two different conduit sizes (18 mm and 26 mm) are displayed. The AVB induced a significant reduction in LV peak pressure and TPGs that can be reduced down from 99 mmHg (Fig 2b) to 28/25 mmHg (Fig 3a and 3b) for TPG_max_ and from 52 mmHg (Fig 2b) to 17/15 mmHg (Fig 3a and 3b) for TPG_mean_. Moreover, Fig 3 shows the calculated flow rate distribution between the AS and the AVB. A LV with an AVB with a conduit valve size of 19 mm and a conduit size of 18 mm is expected to eject 36.57% of the total flow rate through the AS and 63.43% through the bypass. Now, if a conduit size of 26 mm is used with the same conduit valve size, flow distribution changes slightly: 40.45% through the AS and 59.55% through the AVB.

### Flow Distribution after AVB

Table 2 represents the flow distribution between the AVB and the aortic valve predicted using the lumped parameter model for EOA = 0.7 cm^2^. Here several possible combinations of conduit valve size and conduit size are considered. For an AS with EOA = 0.7 cm^2^, the percentage of the total flow crossing the aortic valve ranges from 30.57% to 44.53% (mean value: 36%).

### Left Ventricle Stroke Work after AVB

**Fig 4:**
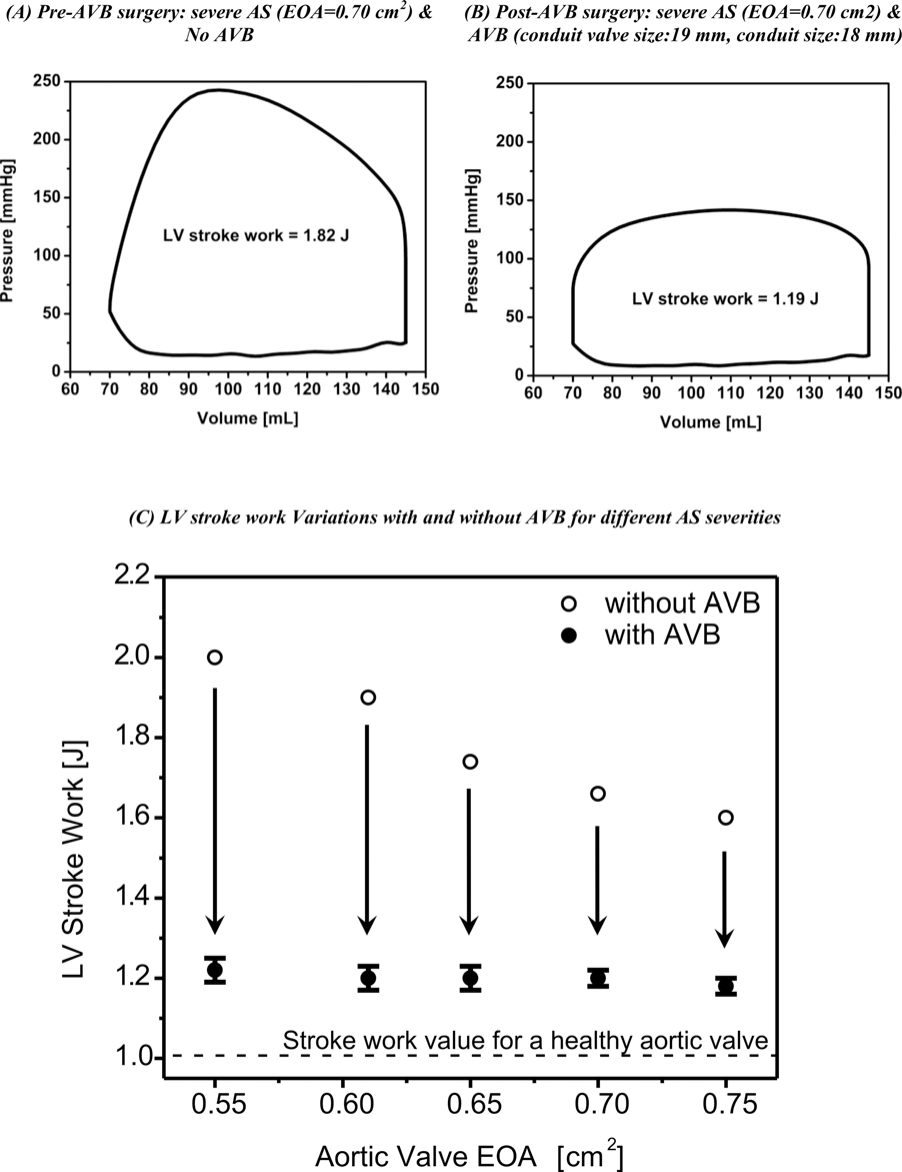
Simulated LV stroke work. (A) Pre-AVB surgery for a severe AS (EOA = 0.7 cm^2^), (B) post-AVB surgery with a conduit valve size of 19 mm and a conduit size of 18mm, (C) LV stroke work variations with and without AVB for different AS severities. The values are averaged over the all configurations for AVB in terms of conduit and valves sizes simulated in this study.

Fig 4 shows the reduction in LV stroke work following the AVB surgery. The LV has to develop a stroke work of 1.82 J in order to overcome the overload imposed by a severe AS with an EOA of 0.7 cm^2^ (Fig 4a). Now with an AVB with a conduit valve size of 19 mm and a conduit size of 18 mm, the LV stroke work is significantly reduced to 1.19 J (-0.63 J; -34%) (Fig 4b). A larger conduit size of 26 mm leads to small variations in LV stroke work compared to a 18 mm conduit (1.21 J *vs*.1.19 J).

Fig 4c shows the reduction in LV stroke work following the AVB surgery for several AS severities (EOA range: 0.55 cm^2^ to 0.75 cm^2^). LV stroke work values corresponding to the AVB for each AS severity were averaged over all configurations of conduit valve size and conduit size tested in this study. For all AS severities, the AVB contributed to a significant reduction in the LV stroke work. The LV stroke work values reached following the AVB surgery are not significantly affected by the selected conduit valve size/conduit size configurations for the AVB and reached a value around 1.2 J. This value remains however higher than normal LV stroke work values (1.03 J).

## Discussion

We have introduced a lumped-parameter method which captures the interaction between left ventricle, AS, arterial system, and AVB. This method only needs few non-invasively measured quantities described as follows: 1) total stroke volume; 2) EOA of the aortic valve and aortic cross sectional area (A); (3) EOA of the conduit valve and conduit area. All the above mentioned parameters can easily be measured in patients non-invasively [17,22]. The key findings of this study are the followings: 1) the AVB leads to a significant reduction in transvalvular pressure gradient; 2) the conduit valve size, compared to the conduit size, has more effect on flow distribution between the AS and the AVB; 3) The flow distribution between the AS and the AVB can be predicted mathematically using non-invasive patient data; 4) the AVB leads to a significant reduction in LV stroke work.

AVB is not a new technique, the first reference to AVB was in 1910 [23] and in 1995 [24], used in animals. This was also followed by its implantation in human by Templeton in 1962 (unpublished work). However, as its development was in the same period of prosthetic heart valves, the success of the AVR reduced temporarily the interests in the AVB. But, with the expected increasing number of high-risk patients with severe symptomatic AS and contraindication to the AVR (due to comorbidities, calcified aorta or narrow LVOT), alternative strategies have to be developed or revived.

TAVI seems to be a very attractive option for such inoperable patients. However, TAVI may be complicated if performed in patients with calcified aorta (expect for the apical approach), ostial encroachment or bicuspid valve. In a recent study, Garcia et al. [25] showed that around 25% of patients with AS have a bicuspid valve. As a consequence, there will always exist a sub-population of ‘no-option’ patients. For such patients, the AVB, using the new developed device by Correx Inc., can be an interesting option for such patients since the aortic valve and the aorta remain undisturbed. As a consequence, this limits significantly the risks of stroke and thromboembolism [10,26]. Furthermore, since the prosthetic valve is located inside the conduit, it is not subjected to prosthesis-patient mismatch restrictions [27].

In this study, we showed that the AVB leads to a significant reduction in TPG. It should be mentioned however that the reduction in the TPG can mainly be attributed to the significant reduction in the flow rate crossing the aortic valve following the AVB surgery.

This flow distribution is a major determinant of the residual transvalvular pressure gradient through the native aortic valve following AVB. To determine this ratio, the model developed in this study needs only non-invasive parameters that are routinely measured in daily clinical practice and the EOA of the bioprosthetic valve in the conduit (listed in Pibarot and Dumesnil [21]). The results, using the current model, show that the flow through the conduit may represent between 55.47% and 72.1% of the total flow rate depending on the EOA of the AS, the EOA of the conduit valve and the size of the conduit. The most important determinant of the flow distribution is the conduit valve size (in this study: R = 0.84, p<0.01). The results obtained in this study are consistent with previous *in vivo* and in silico studies [6,10,26].

Another significant result found in this study is that the AVB significantly reduces the LV stroke work. The expected post-operative LV stroke work is appeared to be quite independent from the selected AVB configurations. Interestingly, it has been hypothesized that the AVB might provide better overall hemodynamic performance since the net effective orifice area should be the summation of the effective orifice area of the stenotic aortic valve plus the effective orifice area of the bioprosthetic valve included in the conduit [5]. Our results show that the LV stroke work values obtained following the AVB surgery are statistically significantly higher than those expected by simply adding up the conduit valve EOA and the aortic valve EOA (1.19±0.03 J *vs*. 1.10±0.05 J, p<0.001). However, the differences appear not to be clinically significant.

## Limitations

Since no formulation has been developed to express the net instantaneous pressure gradient through a conduit valve yet, we elected to use the same formulation as the one used for modeling the aortic valve. In order to assess the validity of this formulation, data resulting from our mathematical simulations were compared with in vivo findings (Stauffer et al. [14]), resulted to good agreements between them (range of errors: 0.11% to 8.46%). However, more *in vivo* and *in vivo* studies are still required to determine further the validity of the equation mainly for large conduit valve sizes.

## Conclusions

AVB is a viable solution for patients with AS and contraindications to both AVR and TAVI. AVB leads to a double outlet LV. In this study, we have shown by using mathematical modelling that AVB leads to a significant reduction in pressure gradient across the AS and LV stroke work. Furthermore, the results show that the flow distribution (through AS *vs*. AVB) can be predicted mathematically and its main determinant is conduit valve size. Finally, AVB leads a significant reduction in LV stroke work whatever is the configuration of conduit valve size and conduit size. Finally, the positive hemodynamic results obtained in this study following AVB may suggest a need for a randomized trial comparing TAVI and AVB.
